# Supplementary material for: A systematic review and embryological perspective of pluripotent stem cell-derived autonomic postganglionic neuron differentiation for human disease modeling
Source: eLife. 2025 Mar 12;14:e103728. doi: 10.7554/eLife.103728 (PMC11961123; doi:10.7554/eLife.103728)
Supplement: Supplementary file 1. — A list of all articles that were excluded during full-text screening, including the reason of exclusion. PSCs, pluripotent stem cells. [file elife-103728-supp1.docx]

# Supplementary File 1 – List of excluded articles

*PSCs, Pluripotent stem cells.*

| **Authors (Year of publication)** | **Title** | **First exclusion reason** |
| --- | --- | --- |
| Birren & Anderson (1990)^1^ | A v-myc-immortalized sympathoadrenal progenitor cell line in which neuronal differentiation is initiated by FGF but not NGF | Non-human |
| Birren et al. (1992)^2^ | Membrane depolarization induces p140trk and NGF responsiveness, but not p75LNGFR, in MAH cells | Non-human |
| Ip et al. (1994)^3^ | CNTF, FGF, and NGF collaborate to drive the terminal differentiation of MAH cells into postmitotic neurons | Non-human |
| Verdi et al. (1994)^4^ | Expression of trk in MAH cells lacking the p75 low-affinity nerve growth factor receptor is sufficient to permit nerve growth factor-induced differentiation to postmitotic neurons | Non-human |
| Doering et al. (1995)^5^ | Ciliary neurotrophic factor promotes the terminal differentiation of v-myc immortalized sympathoadrenal progenitor cells in vivo | Non-human |
| Sommer et al. (1995)^6^ | The cellular function of MASH1 in autonomic neurogenesis | Non-human |
| Negro & Skaper (1996)^7^ | Synthesis, cytotoxic properties and effects on early and late gene induction of a chimeric diphtheria toxin-leukemia-inhibitory factor protein | No PSCs or immortalization |
| Gaspar et al. (1997)^8^ | Effects of neurotrophic factors and cell substrates on the differentiation of a sympathoadrenal progenitor cell line | Non-human |
| Hill & Robertson (1998)^9^ | Differentiation of LA-N-5 neuroblastoma cells into cholinergic neurons: Methods for differentiation, immunohistochemistry and reporter gene introduction | No autonomic neurons/precursors |
| Song et al. (1998)^10^ | Bone morphogenetic proteins induce apoptosis and growth factor dependence of cultured sympathoadrenal progenitor cells | Non-human |
| Thiel & Cibelli (1999)^11^ | Corticotropin-releasing factor and vasoactive intestinal polypeptide activate gene transcription through the cAMP signaling pathway in a catecholaminergic immortalized neuron | Non-human |
| Bharmal et al. (2001)^12^ | Target cells promote the development and functional maturation of neurons derived from a sympathetic precursor cell line | Non-human |
| Venkatesan et al. (2002)^13^ | Endomorphin-2 inhibits GABAergic inputs to cardiac parasympathetic neurons in the nucleus ambiguus | No PSCs or immortalization |
| Venkatesan et al. (2002)^14^ | Nociceptin inhibits gamma-aminobutyric acidergic inputs to cardiac parasympathetic neurons in the nucleus ambiguus | No PSCs or immortalization |
| Clouthier et al. (2003)^15^ | Cell-autonomous and nonautonomous actions of endothelin-A receptor signaling in craniofacial and cardiovascular development | Non-human |
| Gao et al. (2005)^16^ | Human neural stem cell-derived cholinergic neurons innervate muscle in motoneuron deficient adult rats | No PSCs or immortalization |
| Pomp et al. (2005)^17^ | Generation of peripheral sensory and sympathetic neurons and neural crest cells from human embryonic stem cells | Non-homogenous neuron population |
| Singh Roy et al. (2005)^18^ | Enhancer-specified GFP-based FACS purification of human spinal motor neurons from embryonic stem cells | No autonomic neurons/precursors |
| Gossrau et al. (2007)^19^ | Bone morphogenetic protein-mediated modulation of lineage diversification during neural differentiation of embryonic stem cells | Non-human |
| Johnson et al. (2007)^20^ | Functional neural development from human embryonic stem cells: Accelerated synaptic activity via astrocyte coculture | No autonomic neurons/precursors |
| Erceg et al. (2008)^21^ | Differentiation of human embryonic stem cells to regional specific neural precursors in chemically defined medium conditions | No autonomic neurons/precursors |
| Murata et al. (2008)^22^ | Neuronal differentiation elicited by glial cell line-derived neurotrophic factor and ciliary neurotrophic factor in adrenal chromaffin cell line tsAM5D immortalized with temperature-sensitive SV40 T-antigen | Non-human |
| Hashimoto et al. (2009)^23^ | The bHLH transcription factor Hand2 regulates the expression of nanog in ANS differentiation | No PSCs or immortalization |
| Jiang et al. (2009)^24^ | Isolation and characterization of neural crest stem cells derived from in vitro-differentiated human embryonic stem cells | Non-homogenous neuron population |
| Lee et al. (2009)^25^ | Modelling pathogenesis and treatment of familial dysautonomia using patient-specific iPSCs | No autonomic neurons/precursors |
| Michibata et al. (2009)^26^ | Human GPM6A is associated with differentiation and neuronal migration of neurons derived from human embryonic stem cells | Non-homogenous neuron population |
| Schwarz et al. (2009)^27^ | Neuropilin 1 signaling guides neural crest cells to coordinate pathway choice with cell specification | No PSCs or immortalization |
| Valensi-Kurtz et al. (2010)^28^ | Enriched population of PNS neurons derived from human embryonic stem cells as a platform for studying peripheral neuropathies | Non-homogenous neuron population |
| Lee et al. (2012)^29^ | Large-scale screening using familial dysautonomia induced pluripotent stem cells identifies compounds that rescue IKBKAP expression | No autonomic neurons/precursors |
| Lüningschrör et al. (2012)^30^ | Knockdown of IKK1/2 promotes differentiation of mouse embryonic stem cells into neuroectoderm at the expense of mesoderm | Non-human |
| Vukicevic et al. (2012)^31^ | Modulation of Dopaminergic Neuronal Differentiation from Sympathoadrenal Progenitors | Review article |
| Fujiwara et al. (2013)^32^ | Restoration of spatial memory dysfunction of human APP transgenic mice by transplantation of neuronal precursors derived from human iPS cells | No autonomic neurons/precursors |
| Liu et al. (2013)^33^ | Neural differentiation of human embryonic stem cells as an in vitro tool for the study of the expression patterns of the neuronal cytoskeleton during neurogenesis | No autonomic neurons/precursors |
| Reinhardt et al. (2013)^34^ | Derivation and Expansion Using Only Small Molecules of Human Neural Progenitors for Neurodegenerative Disease Modeling | No autonomic neurons/precursors |
| Efthymiou et al. (2014)^35^ | Functional screening assays with neurons generated from pluripotent stem cell-derived neural stem cells | No autonomic neurons/precursors |
| Liu et al. (2014)^36^ | Human neural crest stem cells derived from human pluripotent stem cells | Non-homogenous neuron population |
| Mong et al. (2014)^37^ | Transcription factor-induced lineage programming of noradrenaline and motor neurons from embryonic stem cells | No autonomic neurons/precursors |
| Tarunina et al. (2014)^38^ | Directed differentiation of embryonic stem cells using a bead-based combinatorial screening method | No autonomic neurons/precursors |
| Acevedo et al. (2015)^39^ | hESC Differentiation toward an Autonomic Neuronal Cell Fate Depends on Distinct Cues from the Co-Patterning Vasculature | Non-homogenous neuron population |
| Begum et al. (2015)^40^ | Rapid generation of sub-type, region-specific neurons and neural networks from human pluripotent stem cell-derived neurospheres | No autonomic neurons/precursors |
| Fujiwara et al. (2015)^41^ | Cellular and molecular mechanisms of the restoration of human APP transgenic mouse cognitive dysfunction after transplant of human iPS cell-derived neural cells | No autonomic neurons/precursors |
| Imaizumi et al. (2015)^42^ | Controlling the Regional Identity of hPSC-Derived Neurons to Uncover Neuronal Subtype Specificity of Neurological Disease Phenotypes | No autonomic neurons/precursors |
| Lee et al. (2015)^43^ | Single Transcription Factor Conversion of Human Blood Fate to NPCs with CNS and PNS Developmental Capacity | No PSCs or immortalization |
| Lefler et al. (2015)^44^ | Familial Dysautonomia (FD) Human Embryonic Stem Cell Derived PNS Neurons Reveal that Synaptic Vesicular and Neuronal Transport Genes Are Directly or Indirectly Affected by IKBKAP Downregulation | Non-homogenous neuron population |
| Markus et al. (2015)^45^ | An In Vitro Model of Latency and Reactivation of Varicella Zoster Virus in Human Stem Cell-Derived Neurons | No autonomic neurons/precursors |
| Miura et al. (2015)^46^ | Generation of primitive neural stem cells from human fibroblasts using a defined set of factors | No PSCs or immortalization |
| Romanyuk et al. (2015)^47^ | Beneficial effect of human induced pluripotent stem cell-derived neural precursors in spinal cord injury repair | No autonomic neurons/precursors |
| Fattahi et al. (2016)^48^ | Deriving human ENS lineages for cell therapy and drug discovery in Hirschsprung disease | No autonomic neurons/precursors |
| Ho et al. (2016)^49^ | Rapid Ngn2-induction of excitatory neurons from hiPSC-derived neural progenitor cells | No autonomic neurons/precursors |
| Takayama & Kida (2016)^50^ | In Vitro Reconstruction of Neuronal Networks Derived from Human iPS Cells Using Microfabricated Devices | No autonomic neurons/precursors |
| Vazquez-Arango et al. (2016)^51^ | Variant U1 snRNAs are implicated in human pluripotent stem cell maintenance and neuromuscular disease | No autonomic neurons/precursors |
| Zhou et al. (2016)^52^ | Generation of Human Embryonic Stem Cell Line Expressing zsGreen in Cholinergic Neurons Using CRISPR/Cas9 System | No autonomic neurons/precursors |
| Course et al. (2017)^53^ | Live imaging mitochondrial transport in neurons | No autonomic neurons/precursors |
| Kim et al. (2017)^54^ | Schwann Cell Precursors from Human Pluripotent Stem Cells as a Potential Therapeutic Target for Myelin Repair | No autonomic neurons/precursors |
| Oh et al. (2017)^55^ | Zika virus directly infects peripheral neurons and induces cell death | No autonomic neurons/precursors |
| Romero-Moya et al. (2017)^56^ | Genetic Rescue of Mitochondrial and Skeletal Muscle Impairment in an Induced Pluripotent Stem Cells Model of Coenzyme Q10 Deficiency | No autonomic neurons/precursors |
| Rubi et al. (2017)^57^ | Modulation of the heart's electrical properties by the anticonvulsant drug retigabine | No PSCs or immortalization |
| Vichier-Guerre et al. (2017)^58^ | Impact of selective serotonin reuptake inhibitors on neural crest stem cell formation | No autonomic neurons/precursors |
| Abu-Bonsrah et al. (2018)^59^ | Generation of Adrenal Chromaffin-like Cells from Human Pluripotent Stem Cells | No autonomic neurons/precursors |
| De Santis et al. (2018)^60^ | Direct conversion of human pluripotent stem cells into cranial motor neurons using a piggyBac vector | No autonomic neurons/precursors |
| Abo-Rady et al. (2019)^61^ | Phenotypic screening using mouse and human stem cell-based models of neuroinflammation and gene expression analysis to study drug responses | No autonomic neurons/precursors |
| Alvarez-Carbonell et al. (2019)^62^ | Cross-talk between microglia and neurons regulates HIV latency | No autonomic neurons/precursors |
| Mohlin & Kerosuo (2019)^63^ | In Vitro Maintenance of Multipotent Neural Crest Stem Cells as Crestospheres | No autonomic neurons/precursors |
| Pellett et al. (2019)^64^ | Botulinum neurotoxins A, B, C, E, and F preferentially enter cultured human motor neurons compared to other cultured human neuronal populations | No autonomic neurons/precursors |
| Garcia-Diaz et al. (2020)^65^ | Standardized Reporter Systems for Purification and Imaging of Human Pluripotent Stem Cell-derived Motor Neurons and Other Cholinergic Cells | No autonomic neurons/precursors |
| Hosseini et al. (2020)^66^ | Differentiation of human embryonic stem cells into neuron, cholinergic, and glial cells | No autonomic neurons/precursors |
| Kim et al. (2020)^67^ | Directly induced human Schwann cell precursors as a valuable source of Schwann cells | No PSCs or immortalization |
| Mennen et al. (2020)^68^ | Oxygen tension influences embryonic stem cell maintenance and has lineage specific effects on neural and cardiac differentiation | Non-human |
| Belair et al. (2021)^69^ | Investigation Into the Role of ERK in Tyrosine Kinase Inhibitor-Induced Neuropathy | No autonomic neurons/precursors |
| Gunaseelan et al. (2021)^70^ | Loss of FEZ1, a gene deleted in Jacobsen syndrome, causes locomotion defects and early mortality by impairing motor neuron development | No autonomic neurons/precursors |
| Jeong et al. (2021)^71^ | Direct SARS-CoV-2 infection of the human inner ear may underlie COVID-19-associated audiovestibular dysfunction | No autonomic neurons/precursors |
| Okuno & Okano (2021)^72^ | Modeling human congenital disorders with neural crest developmental defects using patient-derived induced pluripotent stem cells | Review article |
| Ordureau et al. (2021)^73^ | Temporal proteomics during neurogenesis reveals large-scale proteome and organelle remodeling via selective autophagy | No autonomic neurons/precursors |
| Pandya et al. (2021)^74^ | A non-toxic concentration of telomerase inhibitor BIBR1532 fails to reduce tert expression in a feeder-free induced pluripotent stem cell model of human motor neurogenesis | No autonomic neurons/precursors |
| Solomon et al. (2021)^75^ | Global transcriptome profile of the developmental principles of in vitro iPSC-to-motor neuron differentiation | No autonomic neurons/precursors |
| Togo et al. (2021)^76^ | Postsynaptic structure formation of human iPS cell-derived neurons takes longer than presynaptic formation during neural differentiation in vitro | No autonomic neurons/precursors |
| Wang et al. (2021)^77^ | Mechanisms of peripheral neurotoxicity associated with four chemotherapy drugs using human induced pluripotent stem cell-derived peripheral neurons | No autonomic neurons/precursors |
| Clement et al. (2022)^78^ | Dendritic Polyglycerol Amine: An Enhanced Substrate to Support Long-Term Neural Cell Culture | No autonomic neurons/precursors |
| Cooper & Tsakiridis (2022)^79^ | Shaping axial identity during human pluripotent stem cell differentiation to neural crest cells | No autonomic neurons/precursors |
| Cuomo et al. (2022)^80^ | CellRegMap: a statistical framework for mapping context-specific regulatory variants using scRNA-seq | No autonomic neurons/precursors |
| Gogolou et al. (2022)^81^ | Early anteroposterior regionalisation of human neural crest is shaped by a pro-mesodermal factor | No autonomic neurons/precursors |
| Gonzalez et al. (2022)^82^ | Small molecule modulation of TrkB and TrkC neurotrophin receptors prevents cholinergic neuron atrophy in an Alzheimer's disease mouse model at an advanced pathological stage | No autonomic neurons/precursors |
| Hakli et al. (2022)^83^ | Human Neurons Form Axon-Mediated Functional Connections with Human Cardiomyocytes in Compartmentalized Microfluidic Chip | No autonomic neurons/precursors |
| Lin et al. (2022)^84^ | ETS1 loss in mice impairs cardiac outflow tract septation via a cell migration defect autonomous to the neural crest | No autonomic neurons/precursors |
| Majd et al. (2022)^85^ | Deriving Schwann Cells from hPSCs Enables Disease Modeling and Drug Discovery for Diabetic Peripheral Neuropathy | No autonomic neurons/precursors |
| Miranda et al. (2022)^86^ | A Dynamic 3D Aggregate-Based System for the Successful Expansion and Neural Induction of Human Pluripotent Stem Cells | No autonomic neurons/precursors |
| Chitrangi et al. (2023)^87^ | Patient-derived organoids for precision oncology: a platform to facilitate clinical decision making | No autonomic neurons/precursors |
| Davis-Anderson et al. (2023)^88^ | CRISPR/Cas9 Directed Reprogramming of iPSC for Accelerated Motor Neuron Differentiation Leads to Dysregulation of Neuronal Fate Patterning and Function | No autonomic neurons/precursors |
| Enderami et al. (2023)^89^ | Enhanced yield of cholinergic neurons from induced pluripotent stem cells (iPSC): A two-step induction protocol | No autonomic neurons/precursors |
| Kanno et al. (2023)^90^ | SOCS7-Derived BC-Box Motif Peptide Mediated Cholinergic Differentiation of Human Adipose-Derived Mesenchymal Stem Cells | No PSCs or immortalization |
| Koh et al. (2023)^91^ | Visualization of accessible cholesterol using a GRAM domain-based biosensor | No autonomic neurons/precursors |
| Lamiable et al. (2023)^92^ | Revealing invisible cell phenotypes with conditional generative modeling | No autonomic neurons/precursors |
| Perego et al. (2023)^93^ | Arsenic Impairs Differentiation of Human Induced Pluripotent Stem Cells into Cholinergic Motor Neurons | No autonomic neurons/precursors |
| Rockel et al. (2023)^94^ | Neuro-mesodermal assembloids recapitulate aspects of peripheral nervous system development in vitro | Non-homogenous neuron population |
| Saito-Diaz et al. (2023)^95^ | Genipin Crosslinks the Extracellular Matrix to Rescue Developmental and Degenerative Defects, and Accelerates Regeneration of Peripheral Neurons | No peer-reviewed full text |
| Dermentzaki et al. (2024)^96^ | Depletion of Mettl3 in cholinergic neurons causes adult-onset neuromuscular degeneration | Non-human |
| Mathias et al. (2024)^97^ | Human stem cell derived neurons and astrocytes to detect novel auto-reactive IgG signature in immune-mediated neurological diseases | No peer-reviewed full text |
| Ofrim et al. (2024)^98^ | Characterization of two human induced pluripotent stem cell lines derived from Batten disease patient fibroblasts harbouring CLN5 mutations | No autonomic neurons/precursors |
| Passos et al. (2024)^99^ | Innate immune response to SARS-CoV-2 infection contributes to neuronal damage in human iPSC-derived peripheral neurons | No autonomic neurons/precursors |
| Xue et al. (2024)^100^ | A patterned human neural tube model using microfluidic gradients | Non-homogenous neuron population |
| Patel et al. (2024)^101^ | Establishment of a Serum-Free Human iPSC-Derived Model of Peripheral Myelination | No autonomic neurons/precursors |

# References

1. Birren, S.J., and Anderson, D.J. (1990). A v-myc-immortalized sympathoadrenal progenitor cell line in which neuronal differentiation is initiated by FGF but not NGF. Neuron *4*, 189-201. 10.1016/0896-6273(90)90094-v.

2. Birren, S.J., Verdi, J.M., and Anderson, D.J. (1992). Membrane depolarization induces p140trk and NGF responsiveness, but not p75LNGFR, in MAH cells. Science *257*, 395-397. 10.1126/science.1321502.

3. Ip, N.Y., Boulton, T.G., Li, Y., Verdi, J.M., Birren, S.J., Anderson, D.J., and Yancopoulos, G.D. (1994). CNTF, FGF, and NGF collaborate to drive the terminal differentiation of MAH cells into postmitotic neurons. Neuron *13*, 443-455. 10.1016/0896-6273%2894%2990359-X.

4. Verdi, J.M., Ip, N., Yancopoulos, G.D., and Anderson, D.J. (1994). Expression of trk in MAH cells lacking the p75 low-affinity nerve growth factor receptor is sufficient to permit nerve growth factor-induced differentiation to postmitotic neurons. Proc Natl Acad Sci U S A *91*, 3949-3953. 10.1073/pnas.91.9.3949.

5. Doering, L.C., Roder, J.C., and Henderson, J.T. (1995). Ciliary neurotrophic factor promotes the terminal differentiation of v-myc immortalized sympathoadrenal progenitor cells in vivo. Dev. Brain Res. *89*, 56-66. 10.1016/0165-3806(95)00095-u.

6. Sommer, L., Shah, N., Rao, M., and Anderson, D.J. (1995). The cellular function of MASH1 in autonomic neurogenesis. Neuron *15*, 1245-1258. 10.1016/0896-6273(95)90005-5.

7. Negro, A., and Skaper, S.D. (1996). Synthesis, cytotoxic properties and effects on early and late gene induction of a chimeric diphtheria toxin-leukemia-inhibitory factor protein. Eur. J. Biochem. *241*, 507-515. 10.1111/j.1432-1033.1996.00507.x.

8. Gaspar, E.M., Tokiwa, M.A., and Doering, L.C. (1997). Effects of neurotrophic factors and cell substrates on the differentiation of a sympathoadrenal progenitor cell line. J. Neurocytol. *26*, 407-422. 10.1023/a:1018521121917.

9. Hill, D.P., and Robertson, K.A. (1998). Differentiation of LA-N-5 neuroblastoma cells into cholinergic neurons: Methods for differentiation, immunohistochemistry and reporter gene introduction. Brain Research Protocols *2*, 183-190. 10.1016/S1385-299X%2897%2900041-X.

10. Song, Q., Mehler, M.F., and Kessler, J.A. (1998). Bone morphogenetic proteins induce apoptosis and growth factor dependence of cultured sympathoadrenal progenitor cells. Dev Biol *196*, 119-127. 10.1006/dbio.1998.8847.

11. Thiel, G., and Cibelli, G. (1999). Corticotropin-releasing factor and vasoactive intestinal polypeptide activate gene transcription through the cAMP signaling pathway in a catecholaminergic immortalized neuron. Neurochemistry International *34*, 183-191. 10.1016/S0197-0186%2898%2900086-2.

12. Bharmal, S., Slonimsky, J.D., Mead, J.N., Sampson, C.P.B., Tolkovsky, A.M., Yang, B., Bargman, R., and Birren, S.J. (2001). Target cells promote the development and functional maturation of neurons derived from a sympathetic precursor cell line. Developmental Neuroscience *23*, 153-164. 10.1159/000048707.

13. Venkatesan, P., Wang, J., Evans, C., Irnaten, M., and Mendelowitz, D. (2002). Endomorphin-2 inhibits GABAergic inputs to cardiac parasympathetic neurons in the nucleus ambiguus. Neuroscience *113*, 975-983, Pii s0306-4522(02)00244-0. 10.1016/s0306-4522(02)00244-0.

14. Venkatesan, P., Wang, J.J., Evans, C., Irnaten, M., and Mendelowitz, D. (2002). Nociceptin inhibits gamma-aminobutyric acidergic inputs to cardiac parasympathetic neurons in the nucleus ambiguus. Journal of Pharmacology and Experimental Therapeutics *300*, 78-82. 10.1124/jpet.300.1.78.

15. Clouthier, D.E., Williams, S.C., Hammer, R.E., Richardson, J.A., and Yanagisawa, M. (2003). Cell-autonomous and nonautonomous actions of endothelin-A receptor signaling in craniofacial and cardiovascular development. Dev Biol *261*, 506-519. 10.1016/s0012-1606(03)00128-3.

16. Gao, J., Coggeshall, R.E., Tarasenko, Y.I., and Wu, P. (2005). Human neural stem cell-derived cholinergic neurons innervate muscle in motoneuron deficient adult rats. Neuroscience *131*, 257-262. 10.1016/j.neuroscience.2004.10.033.

17. Pomp, O., Brokhman, I., Ben-Dor, I., Reubinoff, B., and Goldstein, R.S. (2005). Generation of peripheral sensory and sympathetic neurons and neural crest cells from human embryonic stem cells. Stem Cells *23*, 923-930. 10.1634/stemcells.2005-0038.

18. Singh Roy, N., Nakano, T., Xuing, L., Kang, J., Nedergaard, M., and Goldman, S.A. (2005). Enhancer-specified GFP-based FACS purification of human spinal motor neurons from embryonic stem cells. Experimental Neurology *196*, 224-234. 10.1016/j.expneurol.2005.06.021.

19. Gossrau, G., Thiele, J., Konang, R., Schmandt, T., and Brüstle, O. (2007). Bone morphogenetic protein-mediated modulation of lineage diversification during neural differentiation of embryonic stem cells. Stem Cells *25*, 939-949. 10.1634/stemcells.2006-0299.

20. Johnson, M.A., Weick, J.P., Pearce, R.A., and Zhang, S.C. (2007). Functional neural development from human embryonic stem cells: Accelerated synaptic activity via astrocyte coculture. Journal of Neuroscience *27*, 3069-3077. 10.1523/jneurosci.4562-06.2007.

21. Erceg, S., Lainez, S., Ronaghi, M., Stojkovic, P., Perez-Arago, M.A., Moreno-Manzano, V., Moreno-Palanques, R., Planells-Cases, R., and Stojkovic, M. (2008). Differentiation of human embryonic stem cells to regional specific neural precursors in chemically defined medium conditions. PLoS ONE *3*, e2122. 10.1371/journal.pone.0002122.

22. Murata, T., Tsuboi, M., Koide, N., Hikita, K., Kohno, S., and Kaneda, N. (2008). Neuronal differentiation elicited by glial cell line-derived neurotrophic factor and ciliary neurotrophic factor in adrenal chromaffin cell line tsAM5D immortalized with temperature-sensitive SV40 T-antigen. Journal of Neuroscience Research *86*, 1694-1710. 10.1002/jnr.21632.

23. Hashimoto, Y., Myojin, R., Katoh, N., Ohtsu, M., Tashiro, F., Onoda, F., and Murakami, Y. (2009). The bHLH transcription factor Hand2 regulates the expression of nanog in ANS differentiation. Biochemical and Biophysical Research Communications *390*, 223-229. 10.1016/j.bbrc.2009.09.090.

24. Jiang, X., Gwye, Y., McKeown, S.J., Bronner-Fraser, M., Lutzko, C., and Lawlor, E.R. (2009). Isolation and characterization of neural crest stem cells derived from in vitro-differentiated human embryonic stem cells. Stem Cells Dev *18*, 1059-1070. 10.1089/scd.2008.0362.

25. Lee, G., Papapetrou, E.P., Kim, H., Chambers, S.M., Tomishima, M.J., Fasano, C.A., Ganat, Y.M., Menon, J., Shimizu, F., Viale, A., et al. (2009). Modelling pathogenesis and treatment of familial dysautonomia using patient-specific iPSCs. Nature *461*, 402-406. 10.1038/nature08320.

26. Michibata, H., Okuno, T., Konishi, N., Kyono, K., Wakimoto, K., Aoki, K., Kondo, Y., Takata, K., Kitamura, Y., and Taniguchi, T. (2009). Human GPM6A is associated with differentiation and neuronal migration of neurons derived from human embryonic stem cells. Stem Cells and Development *18*, 629-639. 10.1089/scd.2008.0215.

27. Schwarz, Q., Maden, C.H., Vieira, J.M., and Ruhrberg, C. (2009). Neuropilin 1 signaling guides neural crest cells to coordinate pathway choice with cell specification. Proceedings of the National Academy of Sciences of the United States of America *106*, 6164-6169. 10.1073/pnas.0811521106.

28. Valensi-Kurtz, M., Lefler, S., Cohen, M.A., Aharonowiz, M., Cohen-Kupiec, R., Sheinin, A., Ashery, U., Reubinoff, B., and Weil, M. (2010). Enriched population of PNS neurons derived from human embryonic stem cells as a platform for studying peripheral neuropathies. PLoS One *5*, e9290. 10.1371/journal.pone.0009290.

29. Lee, G., Ramirez, C.N., Kim, H., Zeltner, N., Liu, B., Radu, C., Bhinder, B., Kim, Y.J., Choi, I.Y., Mukherjee-Clavin, B., et al. (2012). Large-scale screening using familial dysautonomia induced pluripotent stem cells identifies compounds that rescue IKBKAP expression. Nat Biotechnol *30*, 1244-1248. 10.1038/nbt.2435.

30. Lüningschrör, P., Kaltschmidt, B., and Kaltschmidt, C. (2012). Knockdown of IKK1/2 promotes differentiation of mouse embryonic stem cells into neuroectoderm at the expense of mesoderm. Stem Cell Rev Rep *8*, 1098-1108. 10.1007/s12015-012-9402-7.

31. Vukicevic, V., de Celis, M.F.R., Diaz-Valencia, G., Bornstein, S.R., and Ehrhart-Bornstein, M. (2012). Modulation of Dopaminergic Neuronal Differentiation from Sympathoadrenal Progenitors. Journal of Molecular Neuroscience *48*, 420-426. 10.1007/s12031-012-9746-0.

32. Fujiwara, N., Shimizu, J., Takai, K., Arimitsu, N., Saito, A., Kono, T., Umehara, T., Ueda, Y., Wakisaka, S., Suzuki, T., and Suzuki, N. (2013). Restoration of spatial memory dysfunction of human APP transgenic mice by transplantation of neuronal precursors derived from human iPS cells. Neuroscience Letters *557*, 129-134. 10.1016/j.neulet.2013.10.043.

33. Liu, C., Zhong, Y., Apostolou, A., and Fang, S. (2013). Neural differentiation of human embryonic stem cells as an in vitro tool for the study of the expression patterns of the neuronal cytoskeleton during neurogenesis. Biochem Biophys Res Commun *439*, 154-159. 10.1016/j.bbrc.2013.07.130.

34. Reinhardt, P., Glatza, M., Hemmer, K., Tsytsyura, Y., Thiel, C.S., Hoing, S., Moritz, S., Parga, J.A., Wagner, L., Bruder, J.M., et al. (2013). Derivation and Expansion Using Only Small Molecules of Human Neural Progenitors for Neurodegenerative Disease Modeling. PLoS ONE *8*, e59252. 10.1371/journal.pone.0059252.

35. Efthymiou, A., Shaltouki, A., Steiner, J.P., Jha, B., Heman-Ackah, S.M., Swistowski, A., Zeng, X., Rao, M.S., and Malik, N. (2014). Functional screening assays with neurons generated from pluripotent stem cell-derived neural stem cells. Journal of Biomolecular Screening *19*, 32-43. 10.1177/1087057113501869.

36. Liu, Q., Swistowski, A., and Zeng, X. (2014). Human neural crest stem cells derived from human pluripotent stem cells. Methods Mol Biol *1210*, 79-90. 10.1007/978-1-4939-1435-7_7.

37. Mong, J., Panman, L., Alekseenko, Z., Kee, N., Stanton, L.W., Ericson, J., and Perlmann, T. (2014). Transcription factor-induced lineage programming of noradrenaline and motor neurons from embryonic stem cells. Stem Cells *32*, 609-622. 10.1002/stem.1585.

38. Tarunina, M., Hernandez, D., Johnson, C.J., Rybtsov, S., Ramathas, V., Jeyakumar, M., Watson, T., Hook, L., Medvinsky, A., Mason, C., and Choo, Y. (2014). Directed differentiation of embryonic stem cells using a bead-based combinatorial screening method. PLoS ONE *9*, e104301. 10.1371/journal.pone.0104301.

39. Acevedo, L.M., Lindquist, J.N., Walsh, B.M., Sia, P., Cimadamore, F., Chen, C., Denzel, M., Pernia, C.D., Ranscht, B., Terskikh, A., et al. (2015). hESC Differentiation toward an Autonomic Neuronal Cell Fate Depends on Distinct Cues from the Co-Patterning Vasculature. Stem Cell Reports *4*, 1075-1088. 10.1016/j.stemcr.2015.04.013.

40. Begum, A.N., Guoynes, C., Cho, J., Hao, J., Lutfy, K., and Hong, Y. (2015). Rapid generation of sub-type, region-specific neurons and neural networks from human pluripotent stem cell-derived neurospheres. Stem Cell Research *15*, 731-741. 10.1016/j.scr.2015.10.014.

41. Fujiwara, N., Shimizu, J., Takai, K., Arimitsu, N., Ueda, Y., Wakisaka, S., Suzuki, T., and Suzuki, N. (2015). Cellular and molecular mechanisms of the restoration of human APP transgenic mouse cognitive dysfunction after transplant of human iPS cell-derived neural cells. Experimental Neurology *271*, 423-431. 10.1016/j.expneurol.2015.07.008.

42. Imaizumi, K., Sone, T., Ibata, K., Fujimori, K., Yuzaki, M., Akamatsu, W., and Okano, H. (2015). Controlling the Regional Identity of hPSC-Derived Neurons to Uncover Neuronal Subtype Specificity of Neurological Disease Phenotypes. Stem Cell Reports *5*, 1010-1022. 10.1016/j.stemcr.2015.10.005.

43. Lee, J.H., Mitchell, R.R., McNicol, J.D., Shapovalova, Z., Laronde, S., Tanasijevic, B., Milsom, C., Casado, F., Fiebig-Comyn, A., Collins, T.J., et al. (2015). Single Transcription Factor Conversion of Human Blood Fate to NPCs with CNS and PNS Developmental Capacity. Cell Reports *11*, 1367-1376. 10.1016/j.celrep.2015.04.056.

44. Lefler, S., Cohen, M.A., Kantor, G., Cheishvili, D., Even, A., Birger, A., Turetsky, T., Gil, Y., Even-Ram, S., Aizenman, E., et al. (2015). Familial Dysautonomia (FD) Human Embryonic Stem Cell Derived PNS Neurons Reveal that Synaptic Vesicular and Neuronal Transport Genes Are Directly or Indirectly Affected by IKBKAP Downregulation. PLoS One *10*, e0138807. 10.1371/journal.pone.0138807.

45. Markus, A., Lebenthal-Loinger, I., Yang, I.H., Kinchington, P.R., and Goldstein, R.S. (2015). An in vitro model of latency and reactivation of varicella zoster virus in human stem cell-derived neurons. PLoS Pathog *11*, e1004885. 10.1371/journal.ppat.1004885.

46. Miura, T., Sugawara, T., Fukuda, A., Tamoto, R., Kawasaki, T., Umezawa, A., and Akutsu, H. (2015). Generation of primitive neural stem cells from human fibroblasts using a defined set of factors. Biology Open *4*, 1595-1607. 10.1242/bio.013151.

47. Romanyuk, N., Amemori, T., Turnovcova, K., Prochazka, P., Onteniente, B., Sykova, E., and Jendelova, P. (2015). Beneficial effect of human induced pluripotent stem cell-derived neural precursors in spinal cord injury repair. Cell Transplantation *24*, 1781-1797. 10.3727/096368914X684042.

48. Fattahi, F., Steinbeck, J.A., Kriks, S., Tchieu, J., Zimmer, B., Kishinevsky, S., Zeltner, N., Mica, Y., El-Nachef, W., Zhao, H., et al. (2016). Deriving human ENS lineages for cell therapy and drug discovery in Hirschsprung disease. Nature *531*, 105-109. 10.1038/nature16951.

49. Ho, S.M., Hartley, B.J., Tcw, J., Beaumont, M., Stafford, K., Slesinger, P.A., and Brennand, K.J. (2016). Rapid Ngn2-induction of excitatory neurons from hiPSC-derived neural progenitor cells. Methods *101*, 113-124. 10.1016/j.ymeth.2015.11.019.

50. Takayama, Y., and Kida, Y.S. (2016). In Vitro Reconstruction of Neuronal Networks Derived from Human iPS Cells Using Microfabricated Devices. PLoS One *11*, e0148559. 10.1371/journal.pone.0148559.

51. Vazquez-Arango, P., Vowles, J., Browne, C., Hartfield, E., Fernandes, H.J.R., Mandefro, B., Sareen, D., James, W., Wade-Martins, R., Cowley, S.A., et al. (2016). Variant U1 snRNAs are implicated in human pluripotent stem cell maintenance and neuromuscular disease. Nucleic Acids Research *44*, 10960-10973. 10.1093/nar/gkw711.

52. Zhou, J., Wang, C., Zhang, K., Gong, X., Wang, Y., Li, S., and Luo, Y. (2016). Generation of Human Embryonic Stem Cell Line Expressing zsGreen in Cholinergic Neurons Using CRISPR/Cas9 System. Neurochemical Research *41*, 2065-2074. 10.1007/s11064-016-1918-9.

53. Course, M.M., Hsieh, C.H., Tsai, P.I., Codding-Bui, J.A., Shaltouki, A., and Wang, X. (2017). Live imaging mitochondrial transport in neurons. Neuromethods *123*, 49-66. 10.1007/978-1-4939-6890-9_3.

54. Kim, H.S., Lee, J., Lee, D.Y., Kim, Y.D., Kim, J.Y., Lim, H.J., Lim, S., and Cho, Y.S. (2017). Schwann Cell Precursors from Human Pluripotent Stem Cells as a Potential Therapeutic Target for Myelin Repair. Stem Cell Reports *8*, 1714-1726. 10.1016/j.stemcr.2017.04.011.

55. Oh, Y., Zhang, F.R., Wang, Y.Q., Lee, E.M., Choi, I.Y., Lim, H., Mirakhori, F., Li, R.H., Huang, L.X., Xu, T.L., et al. (2017). Zika virus directly infects peripheral neurons and induces cell death. Nature Neuroscience *20*, 1209-+. 10.1038/nn.4612.

56. Romero-Moya, D., Santos-Ocana, C., Castano, J., Garrabou, G., Rodriguez-Gomez, J.A., Ruiz-Bonilla, V., Bueno, C., Gonzalez-Rodriguez, P., Giorgetti, A., Perdiguero, E., et al. (2017). Genetic Rescue of Mitochondrial and Skeletal Muscle Impairment in an Induced Pluripotent Stem Cells Model of Coenzyme Q<inf>10</inf> Deficiency. Stem Cells *35*, 1687-1703. 10.1002/stem.2634.

57. Rubi, L., Kovar, M., Zebedin-Brandl, E., Koenig, X., Dominguez-Rodriguez, M., Todt, H., Kubista, H., Boehm, S., and Hilber, K. (2017). Modulation of the heart's electrical properties by the anticonvulsant drug retigabine. Toxicol Appl Pharmacol *329*, 309-317. 10.1016/j.taap.2017.06.018.

58. Vichier-Guerre, C., Parker, M., Pomerantz, Y., Finnell, R.H., and Cabrera, R.M. (2017). Impact of selective serotonin reuptake inhibitors on neural crest stem cell formation. Toxicol Lett *281*, 20-25. 10.1016/j.toxlet.2017.08.012.

59. Abu-Bonsrah, K.D., Zhang, D., Bjorksten, A.R., Dottori, M., and Newgreen, D.F. (2018). Generation of Adrenal Chromaffin-like Cells from Human Pluripotent Stem Cells. Stem Cell Reports *10*, 134-150. 10.1016/j.stemcr.2017.11.003.

60. De Santis, R., Garone, M.G., Pagani, F., de Turris, V., Di Angelantonio, S., and Rosa, A. (2018). Direct conversion of human pluripotent stem cells into cranial motor neurons using a piggyBac vector. Stem Cell Research *29*, 189-196. 10.1016/j.scr.2018.04.012.

61. Abo-Rady, M., Bellmann, J., Glatza, M., Marrone, L., Reinhardt, L., Tena, S., and Sterneckert, J. (2019). Phenotypic screening using mouse and human stem cell-based models of neuroinflammation and gene expression analysis to study drug responses. Methods in Molecular Biology *1888*, 21-43. 10.1007/978-1-4939-8891-4_2.

62. Alvarez-Carbonell, D., Ye, F., Ramanath, N., Garcia-Mesa, Y., Knapp, P.E., Hauser, K.F., and Karn, J. (2019). Cross-talk between microglia and neurons regulates HIV latency. PLoS Pathogens *15*, e1008249. 10.1371/journal.ppat.1008249.

63. Mohlin, S., and Kerosuo, L. (2019). In Vitro Maintenance of Multipotent Neural Crest Stem Cells as Crestospheres. Methods Mol Biol *2002*, 1-11. 10.1007/7651_2018_180.

64. Pellett, S., Tepp, W.H., and Johnson, E.A. (2019). Botulinum neurotoxins A, B, C, E, and F preferentially enter cultured human motor neurons compared to other cultured human neuronal populations. FEBS Letters *593*, 2675-2685. 10.1002/1873-3468.13508.

65. Garcia-Diaz, A., Efe, G., Kabra, K., Patel, A., Lowry, E.R., Shneider, N.A., Corneo, B., and Wichterle, H. (2020). Standardized Reporter Systems for Purification and Imaging of Human Pluripotent Stem Cell-derived Motor Neurons and Other Cholinergic Cells. Neuroscience *450*, 48-56. 10.1016/j.neuroscience.2020.06.028.

66. Hosseini, K., Lekholm, E., Ahemaiti, A., and Fredriksson, R. (2020). Differentiation of human embryonic stem cells into neuron, cholinergic, and glial cells. Stem Cells International *2020*, 8827874. 10.1155/2020/8827874.

67. Kim, H.S., Kim, J.Y., Song, C.L., Jeong, J.E., and Cho, Y.S. (2020). Directly induced human Schwann cell precursors as a valuable source of Schwann cells. Stem Cell Res Ther *11*, 257. 10.1186/s13287-020-01772-x.

68. Mennen, R.H., de Leeuw, V.C., and Piersma, A.H. (2020). Oxygen tension influences embryonic stem cell maintenance and has lineage specific effects on neural and cardiac differentiation. Differentiation *115*, 1-10. 10.1016/j.diff.2020.07.001.

69. Belair, D.G., Sudak, K., Connelly, K., Collins, N.D., Kopytek, S.J., and Kolaja, K.L. (2021). Investigation Into the Role of ERK in Tyrosine Kinase Inhibitor-Induced Neuropathy. Toxicol Sci *181*, 160-174. 10.1093/toxsci/kfab033.

70. Gunaseelan, S., Wang, Z., Tong, V.K.J., Ming, S.W.S., Razar, R.B.B.A., Srimasorn, S., Ong, W.Y., Lim, K.L., and Chua, J.J.E. (2021). Loss of FEZ1, a gene deleted in Jacobsen syndrome, causes locomotion defects and early mortality by impairing motor neuron development. Human Molecular Genetics *30*, 5-20. 10.1093/hmg/ddaa281.

71. Jeong, M., Ocwieja, K.E., Han, D., Wackym, P.A., Zhang, Y., Brown, A., Moncada, C., Vambutas, A., Kanne, T., Crain, R., et al. (2021). Direct SARS-CoV-2 infection of the human inner ear may underlie COVID-19-associated audiovestibular dysfunction. Commun Med (Lond) *1*, 44. 10.1038/s43856-021-00044-w.

72. Okuno, H., and Okano, H. (2021). Modeling human congenital disorders with neural crest developmental defects using patient-derived induced pluripotent stem cells. Regen Ther *18*, 275-280. 10.1016/j.reth.2021.08.001.

73. Ordureau, A., Kraus, F., Zhang, J., An, H., Park, S., Ahfeldt, T., Paulo, J.A., and Harper, J.W. (2021). Temporal proteomics during neurogenesis reveals large-scale proteome and organelle remodeling via selective autophagy. Molecular Cell *81*, 5082-5098.e5011. 10.1016/j.molcel.2021.10.001.

74. Pandya, V.A., Crerar, H., Mitchell, J.S., and Patani, R. (2021). A non-toxic concentration of telomerase inhibitor BIBR1532 fails to reduce tert expression in a feeder-free induced pluripotent stem cell model of human motor neurogenesis. International Journal of Molecular Sciences *22 (6)*, 3256. 10.3390/ijms22063256.

75. Solomon, E., Davis-Anderson, K., Hovde, B., Micheva-Viteva, S., Harris, J.F., Twary, S., and Iyer, R. (2021). Global transcriptome profile of the developmental principles of in vitro iPSC-to-motor neuron differentiation. BMC Molecular and Cell Biology *22(1)*, 13. 10.1186/s12860-021-00343-z.

76. Togo, K., Fukusumi, H., Shofuda, T., Ohnishi, H., Yamazaki, H., Hayashi, M.K., Kawasaki, N., Takei, N., Nakazawa, T., Saito, Y., et al. (2021). Postsynaptic structure formation of human iPS cell-derived neurons takes longer than presynaptic formation during neural differentiation in vitro. Molecular Brain *14(1)*, 149. 10.1186/s13041-021-00851-1.

77. Wang, M., Wang, J., Tsui, A.Y.P., Li, Z., Zhang, Y., Zhao, Q., Xing, H., and Wang, X. (2021). Mechanisms of peripheral neurotoxicity associated with four chemotherapy drugs using human induced pluripotent stem cell-derived peripheral neurons. Toxicol In Vitro *77*, 105233. 10.1016/j.tiv.2021.105233.

78. Clement, J.P., Al-Alwan, L., Glasgow, S.D., Stolow, A., Ding, Y., Quevedo Melo, T., Khayachi, A., Liu, Y., Hellmund, M., Haag, R., et al. (2022). Dendritic Polyglycerol Amine: An Enhanced Substrate to Support Long-Term Neural Cell Culture. ASN Neuro *14*, 17590914211073276. 10.1177/17590914211073276.

79. Cooper, F., and Tsakiridis, A. (2022). Shaping axial identity during human pluripotent stem cell differentiation to neural crest cells. Biochem Soc Trans *50*, 499-511. 10.1042/bst20211152.

80. Cuomo, A.S.E., Heinen, T., Vagiaki, D., Horta, D., Marioni, J.C., and Stegle, O. (2022). CellRegMap: a statistical framework for mapping context-specific regulatory variants using scRNA-seq. Mol Syst Biol *18*, e10663. 10.15252/msb.202110663.

81. Gogolou, A., Souilhol, C., Granata, I., Wymeersch, F.J., Manipur, I., Wind, M., Frith, T.J.R., Guarini, M., Bertero, A., Bock, C., et al. (2022). Early anteroposterior regionalisation of human neural crest is shaped by a pro-mesodermal factor. Elife *11*, e74263. 10.7554/eLife.74263.

82. Gonzalez, S., McHugh, T.L.M., Yang, T., Syriani, W., Massa, S.M., Longo, F.M., and Simmons, D.A. (2022). Small molecule modulation of TrkB and TrkC neurotrophin receptors prevents cholinergic neuron atrophy in an Alzheimer's disease mouse model at an advanced pathological stage. Neurobiology of Disease *162*, 105563. 10.1016/j.nbd.2021.105563.

83. Häkli, M., Jäntti, S., Joki, T., Sukki, L., Tornberg, K., Aalto-Setälä, K., Kallio, P., Pekkanen-Mattila, M., and Narkilahti, S. (2022). Human Neurons Form Axon-Mediated Functional Connections with Human Cardiomyocytes in Compartmentalized Microfluidic Chip. International Journal of Molecular Sciences *23*, 18, 3148. 10.3390/ijms23063148.

84. Lin, L., Pinto, A., Wang, L., Fukatsu, K., Yin, Y., Bamforth, S.D., Bronner, M.E., Evans, S.M., Nie, S., Anderson, R.H., et al. (2022). ETS1 loss in mice impairs cardiac outflow tract septation via a cell migration defect autonomous to the neural crest. Hum Mol Genet *31*, 4217-4227. 10.1093/hmg/ddac174.

85. Majd, H., Amin, S., Ghazizadeh, Z., Cesiulis, A., Arroyo, E., Lankford, K., Farahvashi, S., Chemel, A.K., Okoye, M., Scantlen, M.D., et al. (2022). [Pre-print] Deriving Schwann Cells from hPSCs Enables Disease Modeling and Drug Discovery for Diabetic Peripheral Neuropathy. bioRxiv. *17*. 10.1101/2022.08.16.504209.<https://www.biorxiv.org/content/10.1101/2022.08.16.504209v1>

86. Miranda, C.C., Akenhead, M.L., Silva, T.P., Derr, M.A., Vemuri, M.C., Cabral, J.M.S., and Fernandes, T.G. (2022). A Dynamic 3D Aggregate-Based System for the Successful Expansion and Neural Induction of Human Pluripotent Stem Cells. Frontiers in Cellular Neuroscience *16*, 838217. 10.3389/fncel.2022.838217.

87. Chitrangi, S., Vaity, P., Jamdar, A., and Bhatt, S. (2023). Patient-derived organoids for precision oncology: a platform to facilitate clinical decision making. BMC Cancer *23*, 689. 10.1186/s12885-023-11078-9.

88. Davis-Anderson, K., Micheva-Viteva, S., Solomon, E., Hovde, B., Cirigliano, E., Harris, J., Twary, S., and Iyer, R. (2023). CRISPR/Cas9 Directed Reprogramming of iPSC for Accelerated Motor Neuron Differentiation Leads to Dysregulation of Neuronal Fate Patterning and Function. International Journal of Molecular Sciences *24(22)*, 16161, 16161. 10.3390/ijms242216161.

89. Enderami, S.E., Bojnordi Hatef, M.N., and Pasandi, M.S. (2023). Enhanced yield of cholinergic neurons from induced pluripotent stem cells (iPSC): A two-step induction protocol. Bratisl Lek Listy *124*, 267-272. 10.4149/bll_2023_040.

90. Kanno, H., Matsumoto, S., Yoshizumi, T., Nakahara, K., Shinonaga, M., Kubo, A., Fujii, S., Ishizuka, Y., Tanaka, M., Ichihashi, M., and Murata, H. (2023). SOCS7-Derived BC-Box Motif Peptide Mediated Cholinergic Differentiation of Human Adipose-Derived Mesenchymal Stem Cells. Int J Mol Sci *24*, 2786. 10.3390/ijms24032786.

91. Koh, D.H.Z., Naito, T., Na, M., Yeap, Y.J., Rozario, P., Zhong, F.L., Lim, K.L., and Saheki, Y. (2023). Visualization of accessible cholesterol using a GRAM domain-based biosensor. Nat Commun *14*, 6773. 10.1038/s41467-023-42498-7.

92. Lamiable, A., Champetier, T., Leonardi, F., Cohen, E., Sommer, P., Hardy, D., Argy, N., Massougbodji, A., Del Nery, E., Cottrell, G., et al. (2023). Revealing invisible cell phenotypes with conditional generative modeling. Nat Commun *14*, 6386. 10.1038/s41467-023-42124-6.

93. Perego, M.C., McMichael, B.D., McMurry, N.R., Ventrello, S.W., and Bain, L.J. (2023). Arsenic Impairs Differentiation of Human Induced Pluripotent Stem Cells into Cholinergic Motor Neurons. Toxics *11*, 644. 10.3390/toxics11080644.

94. Rockel, A.F., Wagner, N., Spenger, P., Ergün, S., and Wörsdörfer, P. (2023). Neuro-mesodermal assembloids recapitulate aspects of peripheral nervous system development in vitro. Stem Cell Reports *18*, 1155-1165. 10.1016/j.stemcr.2023.03.012.

95. Saito-Diaz, K., Dietrich, P., Wu, H.F., Sun, X., Patel, A.J., Wzientek, C.G., Prudden, A.R., Boons, G.J., Chen, S., Studer, L., et al. (2023). [Pre-print] Genipin Crosslinks the Extracellular Matrix to Rescue Developmental and Degenerative Defects, and Accelerates Regeneration of Peripheral Neurons. bioRxiv. 10.1101/2023.03.22.533831.<https://www.biorxiv.org/content/10.1101/2023.03.22.533831v1>

96. Dermentzaki, G., Furlan, M., Tanaka, I., Leonardi, T., Rinchetti, P., Passos, P.M.S., Bastos, A., Ayala, Y.M., Hanna, J.H., Przedborski, S., et al. (2024). Depletion of Mettl3 in cholinergic neurons causes adult-onset neuromuscular degeneration. Cell Reports *43(4)* 113999, 113999. 10.1016/j.celrep.2024.113999.

97. Mathias, A., Perriot, S., Jones, S., Canales, M., Bernardvalnet, R., Gimenez, M., Torcida, N., Oberholster, L., Hottinger, A.F., Zekeridou, A., et al. (2024). [Pre-print] Human stem cell derived neurons and astrocytes to detect novel auto-reactive IgG signature in immune-mediated neurological diseases. bioRxiv. *28*. 10.1101/2024.02.26.582006.<https://www.biorxiv.org/content/10.1101/2024.02.26.582006v3>

98. Ofrim, M., Little, D., Nazari, M., Minnis, C.J., Devine, M.J., Mole, S.E., Gissen, P., and Lorvellec, M. (2024). Characterization of two human induced pluripotent stem cell lines derived from Batten disease patient fibroblasts harbouring CLN5 mutations. Stem Cell Res *74*, 103291. 10.1016/j.scr.2023.103291.

99. Passos, V., Henkel, L.M., Wang, J., Zapatero-Belinchón, F.J., Möller, R., Sun, G., Waltl, I., Schneider, T., Wachs, A., Ritter, B., et al. (2024). Innate immune response to SARS-CoV-2 infection contributes to neuronal damage in human iPSC-derived peripheral neurons. J Med Virol *96*, e29455. 10.1002/jmv.29455.

100. Xue, X., Kim, Y.S., Ponce-Arias, A.I., O'Laughlin, R., Yan, R.Z., Kobayashi, N., Tshuva, R.Y., Tsai, Y.H., Sun, S., Zheng, Y., et al. (2024). A patterned human neural tube model using microfluidic gradients. Nature *628*, 391-399. 10.1038/s41586-024-07204-7.

101. Patel, A., Williams, M., Hawkins, K., Gallo, L., Grillo, M., Akanda, N., Guo, X., Lambert, S., and Hickman, J.J. (2024). Establishment of a Serum-Free Human iPSC-Derived Model of Peripheral Myelination. ACS Biomater Sci Eng *10*, 7132-7143. 10.1021/acsbiomaterials.4c01431.
