# Supplementary material for: A systematic review and embryological perspective of pluripotent stem cell-derived autonomic postganglionic neuron differentiation for human disease modeling
Source: eLife. 2025 Mar 12;14:e103728. doi: 10.7554/eLife.103728 (PMC11961123; doi:10.7554/eLife.103728)
Supplement: Supplementary file 2. — Detailed criteria and definitions per judgement per criterion. [file elife-103728-supp2.docx]

# Supplementary File 2 – Quality assessment criteria and definitions

| **Questions** | **Interpretation** |
| --- | --- |
| **Pluripotent stem cells** | |
| **1. Cell source:** Is the source of the pluripotent stem cells provided or referred to? | **Yes:** Information included at least sex and health status of donor.  **Incompletely:** Source provided, but no information on sex and/or health status of donor.  **No**: No source was provided. |
| **2. Pluripotent stem cell induction:** Is the method of inducing pluripotent stem cells described or referred to? | **Yes:** Methods were provided for all cell lines applied.  **Incompletely:** Methods were provided for some, but not all cell lines applied.  **No**: No methods were provided.  **n/a:** Only embryonic stem cells were used. |
| **3. Pluripotency:** Were pluripotency markers tested prior to differentiation? | **Yes:** Two or more markers were tested by immunostaining or flow cytometry.  **Incompletely:** Only one marker was tested, and/or was only tested by quantitative reverse transcriptase polymerase chain reaction.  **No:** No pluripotency marker results were reported. |
| **4. Karyotype:** Were pluripotent stem cells karyotyped prior to differentiation? | **Yes:** Pluripotent stem cells were karyotyped prior to differentiation.  **No:** Pluripotent stem cells were not karyotyped, or karyotyping of pluripotent stem cells was not reported. |
| **Neuronal characterization** | |
| **5. Reproducibility:** Did the authors repeat procedures in at least one other cell line? | **Yes:** All procedures were performed in at least two cell lines from different donors.  **Incompletely:** Some, but not all procedures were performed in at least two cell lines from different donors.  **No:** All procedures were performed in cell lines derived from a single donor. |
| **6. Replicates:** Were quantitative results derived from at least three biological replicates? | **Yes:** All results included at least three independent protocol repetitions.  **Incompletely:** Some, but not all results included at least three independent protocol repetitions.  **No:** No results included at least three independent protocol repetitions. |
| **7. Identity markers:** Were identity markers investigated? | **Yes:** At least one identity marker was investigated at the protein and/or gene expression level.  **No:** No identity markers were investigated at the protein and/or gene expression level. |
| **8. Neurotransmitters:** Was neurotransmitter secretion tested? | **Yes:** Adrenaline, noradrenaline or acetylcholine concentrations after stimulation or spontaneous release were reported as determined by enzyme-linked immunosorbent assay or high-performance liquid chromatography.  **No:** No adrenaline, noradrenaline or acetylcholine concentrations were reported. |
| **9. Electrophysiology:** Was any form of electrophysiological characterization performed? | **Yes:** Patch clamp, multielectrode array or cytosolic [Ca^2+^] imaging data was reported for the latest version of the protocol reported in the article.  **Incompletely:** Patch clamp, multielectrode array or cytosolic [Ca^2+^] imaging data was only reported for an earlier version of the protocol.  **No:** No electrophysiological characterizations were performed. |
| **10. Co-culture:** Was any form of neuronal coupling to target cells investigated? | **Yes:** Co-culture of neurons and at least one other cell type or tissue was characterized.  **No:** No co-culture experiments were reported. |
| **11. Contamination:** Were cells which did not meet neuron definitions characterized? | **Yes:** Identities of cells not meeting neuron definitions were investigated after immortalization or at the final stage of differentiation.  **Incompletely:** Identities of cells not meeting neuron definitions were investigated before the final stage of differentiation.  **No:** Cells not meeting neuron definitions were not investigated. |
| **Reporting and rationale** | |
| **12. Objectives:** Are specific objectives and/or hypotheses defined? | **Yes:** Specific aims and/or falsifiable hypotheses were explicitly reported.  **Incompletely:** Aims and hypotheses were implied (e.g. in descriptions of experiments or by defining gaps in literature), or were vaguely formulated.  **No:** Aims or hypotheses were not mentioned. |
| **13. Methods:** Do the authors describe the procedures in enough detail to allow others to replicate them? | **Yes:** All experimental procedures were reported with sufficient detail to allow competent external parties to exactly replicate experiments, including concentrations, manufacturers, relevant antibody clones and relevant sequences. Alternatively, a publication which met these demands was referred to.  **Incompletely:** General procedures were described, but were (partially) missing crucial details.  **No:** Experiments or protocol modifications were reported without corresponding methods sections or references. |
| **14. Statistics:** Are enough details for statistical methods provided to allow others to check the results? | **Yes:** All statistical tests, including assumptions tested and software used, were reported.  **Incompletely:** Some statistical details were missing, such as assumptions or software.  **No:** Quantitative data were reported without reporting any statistical methods.  **n/a:** No quantitative data was reported. |
| **15. Results:** Were all quantitative outcomes reported with effect size and a measure of precision? | **Yes:** All quantitative data was reported with effect size (as opposed to only p-values or claims of significance) and a measure of precision (e.g. confidence interval, standard error or standard deviation).  **Incompletely:** Not all quantitative data were reported with effect size and measure of precision.  **n/a:** No quantitative data was reported. |
| **16. Discussion:** Did authors comment on any limitations or future perspectives of their study? | **Yes:** Sufficient discussion of all important limitations or multiple future perspectives.  **Incompletely:** Only a brief and/or superficial discussion of limitations or future perspectives was provided, or an important limitation was not mentioned.  **No:** No limitations or future perspectives were discussed. |
| **17. Interpretation:** Did authors explain their results in the context of study objectives, current theory or other relevant studies? | **Yes:** Results were sufficiently placed in context of study objectives, current theory or other relevant studies.  **Incompletely:** Results were only briefly placed in context of study objectives, current theory or other relevant studies.  **No:** Results were not placed in context of study objectives, current theory or other relevant studies. |
| **Ethics** | |
| **18. Ethical statement:** Were ethical board assessment and informed consent procedures for all patient materials described? | **Yes:** Generation of cell lines was approved by an ethical board, or if received from an external party, ethical procedures performed by the external party were reported.  **No:** Ethical board assessment and informed consent procedures were not described. Alternatively, if cells were derived from an external party, procedures performed by the external party were not reported. |
| **19. Declaration of interests:** Did all authors declare whether they had any potential conflicts of interest? | **Yes:** A conflict of interest statement was reported, either per author, or as a blanket statement.  **No:** No conflict of interest statement was reported. |
| **20. Data access:** Do authors provide a statement describing where study data and resources are available? | **Yes:** All data not evident from the article was provided in a supplementary file, or a statement about data availability was provided.  **Incompletely:** Some, but not all study data not evident from the article was provided in a supplementary file. No statement was made about further data availability.  **No:** No additional data or statement about data availability was provided. |
