# Supplementary material for: A systematic review and embryological perspective of pluripotent stem cell-derived autonomic postganglionic neuron differentiation for human disease modeling
Source: eLife. 2025 Mar 12;14:e103728. doi: 10.7554/eLife.103728 (PMC11961123; doi:10.7554/eLife.103728)
Supplement: Supplementary file 3. — Additional culture details per original protocol, relating to Figures 3, 5 and 6. [file elife-103728-supp3.docx]

| **Protocol** | **Duration (days)** | **PSC maintenance medium** | **Differentiation base medium** | **Maturation base medium** | **Substrates** | **Feeder cells** |
| --- | --- | --- | --- | --- | --- | --- |
| **Huang et al. 2016** | 24 | mTeSR1 | StemPro with FGF2 and without Activin A | StemPro with FGF2 and without Activin A | Geltrex | None |
| **Zhang et al. 2016** | 22 | DMEM/F12 with 20% KSR | GMEM with 10% KSR | GMEM with 10% KSR and N2 | Gelatin, ultra-low attachment, PO/LM/FN | MEFs and PA6 |
| **Oh et al. 2016** | 21 | DMEM/F12 with 20% KSR | DMEM with 15% KSR, gradually replaced by DMEM/F12 with N2 | Neurobasal medium with N2 and B27 | Geltrex | MEF-conditioned |
| **Zeltner et al. 2016** | 30 | DMEM/F12 with 20% KSR | DMEM with 15% KSR, gradually replaced by DMEM/F12 with N2 | Neurobasal medium with N2 and B27 | Gelatin, Matrigel, ultra-low attachment, PO/LM/FN | MEFs |
| **Frith et al. 2018** | 18 | Essential 8 or mTeSR1 | DMEM/F12 and neurobasal medium 1:1 with N2 and B27, followed by DMEM/F12 with N2 | BrainPhys with N2 and B27 | Vitronectin or laminin 521, Geltrex | None |
| **Kirino et al. 2018** | 31 | mTeSR1 | Essential 6 | Neurobasal with N2 and B27, DMEM with 10% FBS | Matrigel, ultra-low attachment | None |
| **Carr-Wilkinson et al. 2018** | 22 | KO-DMEM with 20% KSR | MACS neuronal medium with N2 and B27 | MACS neuronal medium with N2 | PA6 substrate | Fed1A or MEFs, and PA6 |
| **Hackland et al. 2019** | 17 | mTeSR1 | DMEM/F12 with N2 | DMEM/F12 with N2 and B27 | Matrigel | None |
| **Gomez et al. 2019** | 9 | mTeSR1 | DMEM/F12 with B27 | DMEM/F12 with N2 and B27 | Matrigel | None |
| **Wu & Zeltner 2020** | 30 | Essential 8 | Essential 6 | Neurobasal medium with N2 and B27 | Vitronectin, Geltrex, ultra-low attachment, PO/LM/FN | None |
| **Winbo et al. 2020** | 48 | Essential 8 or StemFlex | StemFlex, gradually replaced by Neurobasal plus medium with N2 and B27 | Neurobasal plus medium with N2 and B27 | Geltrex | None |
| **Van Haver et al. 2024** | 32 | Essential 8 | Essential 6 | Neurobasal medium with N2 and B27 | Geltrex, ultra-low attachment, PO/LM/FN | None |
| **Fan et al. 2024b** | 42 | mTeSR Plus | DMEM/F12 and neurobasal medium 1:1 with N2, B27, EGF, and FGF2, followed by neurobasal medium with N2 | DMEM/F12 and neurobasal medium 1:1 with N2 and B27 | Matrigel, ultra-low attachment, Matrigel | None |
| **Takayama et al. 2020** | 34 | mTeSR1 | DMEM/F12 with 20% KSR, gradually replaced by DMEM/F12 with N2 | DMEM/F12 with N2 | Laminin 511-E8, MPC, PO/LM | None |
| **Goldsteen et al. 2022** | 50 | mTeSR1 | KO-DMEM with 15% KSR, gradually replaced by DMEM/F12 HEPES with N2 (2x) | Neurobasal medium with N2 and B27 | Matrigel, Anti adherence rinsing solution, PO/LM/FN | None |
| **Wu et al. 2024b** | 30 | Essential 8 | Essential 6 | Neurobasal medium with B27, followed by neurobasal medium with B27 and 1% FBS | Vitronectin, Geltrex, ultra-low attachment, PO/LM/FN | None |

# Supplementary File 3 – Methodological details of included protocols

*Additional culture details of original protocols, in support of* ***Figures 3, 5 and 6****. For simplicity, amino acids, sugars, reducing agents, heparin and antibiotics in medium have not been described.*

*B27, B27 supplement 1x; DMEM, Dulbecco's modified Eagle medium; EGF, Epidermal growth factor; F12, Ham's F-12 Nutrient Mixture; FBS, Fetal bovine serum; Fed1A, Human foreskin fibroblast Ncl1Fed1A cell line; FGF2, Fibroblast growth factor 2; FN, Fibronectin; GMEM, Glasgow modified Eagle’s medium; HEPES, 4-(2-Hydroxyethyl)-1-piperazineethanesulfonic acid; hESC, Human embryonic stem cells; KO-DMEM, KnockOut DMEM; KSR, Knockout serum replacement; LM, Laminin; MACS, Magnetic-activated cell sorting; MEFs, Mitotically inactivated mouse embryonic fibroblasts; MPC, 2-Methacryloyloxyethyl phosphorylcholine; mTeSR, modified Teneille Serum Replacer; N2, N2 supplement 1x; PA6, Mouse PA6 stromal cells; PO, Polyornithine; PSC, Pluripotent stem cell; StemPro, StemPro hESC serum- and feeder-free medium.*
