# Supplementary material for: A systematic review and embryological perspective of pluripotent stem cell-derived autonomic postganglionic neuron differentiation for human disease modeling
Source: eLife. 2025 Mar 12;14:e103728. doi: 10.7554/eLife.103728 (PMC11961123; doi:10.7554/eLife.103728)
Supplement: Supplementary file 4. — Modes of actions for all small molecules featured in this article. [file elife-103728-supp4.docx]

# Supplementary File 4 – Small molecules used in autonomic neuron protocols

| **Small molecule** | **Abbreviation** | **Mode of action** | **References** |
| --- | --- | --- | --- |
| CHIR99021 | CHIR | Indirect activation of Wnt signaling via glycogen synthesis kinase 3 inhibition | ^1^ |
| SB431542 | SB | Inhibition of Activin, Nodal and transforming growth factor beta signaling via inhibition of anaplastic lymphoma kinase (ALK)4, ALK5, and ALK7 receptors | ^2^ |
| LDN193189 | LDN | Inhibition of bone morphogenetic protein signaling via ALK1, ALK2, ALK3 and ALK6 receptor inhibition | ^3^ |
| Dorsomorphin | DMH | Inhibition of bone morphogenetic protein signaling via ALK1, ALK2, ALK3 and ALK6 receptor inhibition | ^4^ |
| DAPT | DAPT | Indirect inhibition of Notch signaling via γ‐secretase inhibition | ^5^ |
| SU5402 | SU5402 | Direct fibroblast growth factor (FGF) receptor 1 and vascular endothelial growth factor receptor inhibition | ^6^ |
| PD173074 | PD173074 | Direct FGF receptor 1 and FGF receptor 3 inhibition | ^7,8^ |
| Purmorphamine | PMP | Activation of Sonic hedgehog signaling via activation of the downstream G-protein coupled receptor Smoothened | ^9^ |
| IWR1 | IWR1 | Indirect inhibition of Wnt signaling via enhanced catenin β 1 degradation | ^10^ |
| SANT1 | SANT1 | Sonic hedgehog inhibition via inhibition of the downstream G-protein coupled receptor Smoothened | ^11^ |
| Dibutyryl cyclic adenosine monophosphate | dbcAMP | Membrane-permeable, stabilized analogue of endogenous cyclic adenosine monophosphate, with skewed isozyme specificity | ^12^ |
| Forskolin | FSK | Enhances intracellular cyclic adenosine monophosphate concentrations by stimulating adenylyl cyclase | ^13^ |

**References**

1. Ring, D.B., Johnson, K.W., Henriksen, E.J., Nuss, J.M., Goff, D., Kinnick, T.R., Ma, S.T., Reeder, J.W., Samuels, I., Slabiak, T., et al. (2003). Selective glycogen synthase kinase 3 inhibitors potentiate insulin activation of glucose transport and utilization in vitro and in vivo. Diabetes *52*, 588-595. 10.2337/diabetes.52.3.588.

2. Inman, G.J., Nicolás, F.J., Callahan, J.F., Harling, J.D., Gaster, L.M., Reith, A.D., Laping, N.J., and Hill, C.S. (2002). SB-431542 is a potent and specific inhibitor of transforming growth factor-beta superfamily type I activin receptor-like kinase (ALK) receptors ALK4, ALK5, and ALK7. Mol Pharmacol *62*, 65-74. 10.1124/mol.62.1.65.

3. Yu, P.B., Deng, D.Y., Lai, C.S., Hong, C.C., Cuny, G.D., Bouxsein, M.L., Hong, D.W., McManus, P.M., Katagiri, T., Sachidanandan, C., et al. (2008). BMP type I receptor inhibition reduces heterotopic [corrected] ossification. Nat Med *14*, 1363-1369. 10.1038/nm.1888.

4. Yu, P.B., Hong, C.C., Sachidanandan, C., Babitt, J.L., Deng, D.Y., Hoyng, S.A., Lin, H.Y., Bloch, K.D., and Peterson, R.T. (2008). Dorsomorphin inhibits BMP signals required for embryogenesis and iron metabolism. Nat Chem Biol *4*, 33-41. 10.1038/nchembio.2007.54.

5. Geling, A., Steiner, H., Willem, M., Bally-Cuif, L., and Haass, C. (2002). A gamma-secretase inhibitor blocks Notch signaling in vivo and causes a severe neurogenic phenotype in zebrafish. EMBO Rep *3*, 688-694. 10.1093/embo-reports/kvf124.

6. Mohammadi, M., McMahon, G., Sun, L., Tang, C., Hirth, P., Yeh, B.K., Hubbard, S.R., and Schlessinger, J. (1997). Structures of the tyrosine kinase domain of fibroblast growth factor receptor in complex with inhibitors. Science *276*, 955-960. 10.1126/science.276.5314.955.

7. Skaper, S.D., Kee, W.J., Facci, L., Macdonald, G., Doherty, P., and Walsh, F.S. (2000). The FGFR1 inhibitor PD 173074 selectively and potently antagonizes FGF-2 neurotrophic and neurotropic effects. J Neurochem *75*, 1520-1527. 10.1046/j.1471-4159.2000.0751520.x.

8. Trudel, S., Ely, S., Farooqi, Y., Affer, M., Robbiani, D.F., Chesi, M., and Bergsagel, P.L. (2004). Inhibition of fibroblast growth factor receptor 3 induces differentiation and apoptosis in t(4;14) myeloma. Blood *103*, 3521-3528. 10.1182/blood-2003-10-3650.

9. Sinha, S., and Chen, J.K. (2006). Purmorphamine activates the Hedgehog pathway by targeting Smoothened. Nat Chem Biol *2*, 29-30. 10.1038/nchembio753.

10. Chen, B., Dodge, M.E., Tang, W., Lu, J., Ma, Z., Fan, C.W., Wei, S., Hao, W., Kilgore, J., Williams, N.S., et al. (2009). Small molecule-mediated disruption of Wnt-dependent signaling in tissue regeneration and cancer. Nat Chem Biol *5*, 100-107. 10.1038/nchembio.137.

11. Chen, J.K., Taipale, J., Young, K.E., Maiti, T., and Beachy, P.A. (2002). Small molecule modulation of Smoothened activity. Proc Natl Acad Sci U S A *99*, 14071-14076. 10.1073/pnas.182542899.

12. Ogreid, D., Ekanger, R., Suva, R.H., Miller, J.P., Sturm, P., Corbin, J.D., and Døskeland, S.O. (1985). Activation of protein kinase isozymes by cyclic nucleotide analogs used singly or in combination. Principles for optimizing the isozyme specificity of analog combinations. Eur J Biochem *150*, 219-227. 10.1111/j.1432-1033.1985.tb09010.x.

13. Alasbahi, R.H., and Melzig, M.F. (2012). Forskolin and derivatives as tools for studying the role of cAMP. Pharmazie *67*, 5-13. 10.1691/ph.2012.1642.
