# Supplementary material for: A systematic review and embryological perspective of pluripotent stem cell-derived autonomic postganglionic neuron differentiation for human disease modeling
Source: eLife. 2025 Mar 12;14:e103728. doi: 10.7554/eLife.103728 (PMC11961123; doi:10.7554/eLife.103728)
Supplement: Supplementary file 5. — Function and expression profiles of molecular autonomic neuron markers. [file elife-103728-supp5.docx]

# Supplementary File 5 – Molecular autonomic neuron markers

| **Molecular marker** | **Abbreviation** | **Function** | **Expression in neurons** |
| --- | --- | --- | --- |
| Tyrosine hydroxylase | **TH** | Conversion of tyrosine to 3,4-di-hydroxy-phenylanine | Sympathetic neurons, embryonic enteric and parasympathetic neurons^1,2^, central nervous system (CNS) catecholaminergic neurons^3^ |
| Peripherin | **PRPH** | Type III intermediate filament | All neurons with peripheral neurites^4^, histaminergic neurons of the tuberomammillary nucleus^5^ |
| Dopamine beta-hydroxylase | **DBH** | Conversion of dopamine to noradrenaline | Sympathetic neurons, embryonic parasympathetic and enteric neurons^1,2^, CNS noradrenergic neurons^3^ |
| Achaete-scute family bHLH transcription factor 1 | **ASCL1** | Transcription factor | Sympathetic neuron precursors, parasympathetic neuron precursors, enteric neuron precursors, CNS neuron precursors^6^ |
| GATA binding protein 3 | **GATA3** | Transcription factor | Sympathetic neurons, cranial ganglia, CNS neurons^7^ |
| Paired like homeobox 2B | **PHOX2B** | Transcription factor | Sympathetic neurons, parasympathetic neurons, enteric neurons, cranial nerve ganglia, CNS noradrenergic neurons^8^ |
| Tubulin beta 3 class III | **TUBB3** | Beta-tubulin isotype | Pan-neuronal^9^ |
| Cholinergic receptor nicotinic alpha 3/beta 4 subunits | **CHRNA3/B4** | Nicotinic acetylcholine receptor subunits | Sympathetic neurons, parasympathetic neurons, enteric neurons, sensory neurons, specific CNS nuclei^10^ |
| Choline O-acetyltransferase | **CHAT** | Synthesis of acetylcholine from acetyl coenzyme A and choline | Parasympathetic neurons, enteric neurons, (preganglionic) spinal motor neurons, embryonic sympathetic neurons, CNS cholinergic neurons^11^ |
| H6 family homeobox 2/3 | **HMX2/3** | Transcription factor | Parasympathetic neurons^12^, enteric neurons^13^, CNS neurons^14^ |

1. Teitelman, G., Baker, H., Joh, T.H., and Reis, D.J. (1979). Appearance of catecholamine-synthesizing enzymes during development of rat sympathetic nervous system: possible role of tissue environment. Proc Natl Acad Sci U S A *76*, 509-513. 10.1073/pnas.76.1.509.

2. Landis, S.C., Jackson, P.C., Fredieu, J.R., and Thibault, J. (1987). Catecholaminergic properties of cholinergic neurons and synapses in adult rat ciliary ganglion. J Neurosci *7*, 3574-3587. 10.1523/jneurosci.07-11-03574.1987.

3. Verney, C. (1999). Distribution of the catecholaminergic neurons in the central nervous system of human embryos and fetuses. Microsc Res Tech *46*, 24-47. 10.1002/(sici)1097-0029(19990701)46:1<24::Aid-jemt3>3.0.Co;2-e.

4. Romano, R., Del Fiore, V.S., and Bucci, C. (2022). Role of the Intermediate Filament Protein Peripherin in Health and Disease. Int J Mol Sci *23*, 15416. 10.3390/ijms232315416.

5. Eriksson, K.S., Zhang, S., Lin, L., Larivière, R.C., Julien, J.P., and Mignot, E. (2008). The type III neurofilament peripherin is expressed in the tuberomammillary neurons of the mouse. BMC Neurosci *9*, 26. 10.1186/1471-2202-9-26.

6. Guillemot, F., Lo, L.C., Johnson, J.E., Auerbach, A., Anderson, D.J., and Joyner, A.L. (1993). Mammalian achaete-scute homolog 1 is required for the early development of olfactory and autonomic neurons. Cell *75*, 463-476. 10.1016/0092-8674(93)90381-y.

7. George, K.M., Leonard, M.W., Roth, M.E., Lieuw, K.H., Kioussis, D., Grosveld, F., and Engel, J.D. (1994). Embryonic expression and cloning of the murine GATA-3 gene. Development *120*, 2673-2686. 10.1242/dev.120.9.2673.

8. Pattyn, A., Morin, X., Cremer, H., Goridis, C., and Brunet, J.F. (1999). The homeobox gene Phox2b is essential for the development of autonomic neural crest derivatives. Nature *399*, 366-370. 10.1038/20700.

9. Katsetos, C.D., Herman, M.M., and Mörk, S.J. (2003). Class III beta-tubulin in human development and cancer. Cell Motil Cytoskeleton *55*, 77-96. 10.1002/cm.10116.

10. Improgo, M.R., Scofield, M.D., Tapper, A.R., and Gardner, P.D. (2010). The nicotinic acetylcholine receptor CHRNA5/A3/B4 gene cluster: dual role in nicotine addiction and lung cancer. Prog Neurobiol *92*, 212-226. 10.1016/j.pneurobio.2010.05.003.

11. Tallini, Y.N., Shui, B., Greene, K.S., Deng, K.Y., Doran, R., Fisher, P.J., Zipfel, W., and Kotlikoff, M.I. (2006). BAC transgenic mice express enhanced green fluorescent protein in central and peripheral cholinergic neurons. Physiol Genomics *27*, 391-397. 10.1152/physiolgenomics.00092.2006.

12. Ernsberger, U., Deller, T., and Rohrer, H. (2020). The diversity of neuronal phenotypes in rodent and human autonomic ganglia. Cell Tissue Res *382*, 201-231. 10.1007/s00441-020-03279-6.

13. Heanue, T.A., and Pachnis, V. (2006). Expression profiling the developing mammalian enteric nervous system identifies marker and candidate Hirschsprung disease genes. Proc Natl Acad Sci U S A *103*, 6919-6924. 10.1073/pnas.0602152103.

14. Wang, W., Lo, P., Frasch, M., and Lufkin, T. (2000). Hmx: an evolutionary conserved homeobox gene family expressed in the developing nervous system in mice and Drosophila. Mech Dev *99*, 123-137. 10.1016/s0925-4773(00)00488-3.
